# Cloning and Characterization of Surface-Localized α-Enolase of *Streptococcus iniae,* an Effective Protective Antigen in Mice

**DOI:** 10.3390/ijms160714490

**Published:** 2015-06-25

**Authors:** Jun Wang, Kaiyu Wang, Defang Chen, Yi Geng, Xiaoli Huang, Yang He, Lili Ji, Tao Liu, Erlong Wang, Qian Yang, Weimin Lai

**Affiliations:** 1 Department of Basic Veterinary, Sichuan Agricultural University, Wenjiang District Huimin Road No. 211, Chengdu 611130, China; E-Mails: wangjunzl@126.com (J.W.); gengyisicau@126.com (Y.G.); heyang@sicau.edu.cn (Y.H.); jllsicau@126.com (L.J.); ltsicau@126.com (T.L.); welsicau@126.com (E.W.); yangqiansicau@126.com (Q.Y.); nwm_mm2004@163.com (W.L.); 2 Key Laboratory of Animal Disease and Human Health of Sichuan Province, Sichuan Agricultural University, Wenjiang District Huimin Road No. 211, Chengdu 611130, China; 3 Department of Aquaculture, Sichuan Agricultural University, Wenjiang District Huimin Road No. 211, Chengdu 611130, China; E-Mails: chendf_sicau@126.com (D.C.); hxldyq@126.com (X.H.)

**Keywords:** *Streptococcus iniae*, α-enolase, characterization, functions, surface location, protective antigen, mice

## Abstract

*Streptococcus iniae* is a major fish pathogen that can also cause human bacteremia, cellulitis and meningitis. Screening for and identification of protective antigens plays an important role in developing therapies against *S. iniae* infections. In this study, we indicated that the α-enolase of *S. iniae* was not only distributed in the cytoplasm and associated to cell walls, but was also secreted to the bacterial cell surface. The functional identity of the purified recombinant α-enolase protein was verified by its ability to catalyze the conversion of 2-phosphoglycerate (2-PGE) to phosphoenolpyruvate (PEP), and both the recombinant and native proteins interacted with human plasminogen. The rabbit anti-rENO serum blockade assay shows that α-enolase participates in *S. iniae* adhesion to and invasion of BHK-21 cells. In addition, the recombinant α-enolase can confer effective protection against *S. iniae* infection in mice, which suggests that α-enolase has potential as a vaccine candidate in mammals. We conclude that *S. iniae* α-enolase is a moonlighting protein that also associates with the bacterial outer surface and functions as a protective antigen in mice.

## 1. Introduction

*Streptococcus iniae* is an aquatic pathogen which has established itself as a zoonotic risk [1,2]. To date, at least 27 human cases of invasive streptococcal infection attributed to *S. iniae* have been reported [1,3,4,5,6,7]. The most common manifestations of *S. iniae* infection are bacteremia cellulitis and meningitis [3], which produce symptoms very similar to those of infections caused by human-specific streptococcal pathogens such as *S. pyogenes*, *S. agalactiae* and *S. pneumonia* [8].

The pathogenesis of disease caused by *S. iniae* is not yet fully understood, however, adhesion and invasion are crucial steps for *S. iniae* to infect hosts and often aided by many surface proteins [9]. Previous data indicated that *S. iniae* was able to survive in serum, and expressed surface factors which could bind trout immune globulin (Ig) [10]. Subsequently, M-like protein was confirmed as a dominant virulence factor, which enables *S. iniae* to adhere to and invade host cells during infection [9]. Additionally, the capacity of adhesion and invasion of epithelial cells were both improved in *S. iniae* without a capsule which increased exposure of surface proteins and also demonstrated that the surface proteins play important roles in *S. iniae* to adhere to and invade host cells [11]. However, due to the complex structure of bacteria, a comprehensive understanding of *S. iniae* surface proteins is still not very clear.

Increasing numbers of reports support the idea that cytoplasmic glycolytic enzymes such as fructose-1, 6-bisphosphate aldolase (FBA), α-enolase and glyceraldehyde-3-phosphate dehydrogenase (GAPDH) can be exported to the cell surface of a variety of prokaryote and eukaryotes, and play a critical role in bacterial adhesion and invasion to host cells [12,13,14,15,16]. The function of α-enolase is to catalyze the reversible conversion of 2-phosphoglycerate into phosphoenolpyruvate when it is present in cytoplasm. Lack of known cell surface protein motifs such as a signal peptidase cleavage site, cell wall anchors or sequences, and membrane spanning domains suggests that the export of α-enolase may depend on covalent binding to the substrate [17]. It has been confirmed in many microorganisms that α-enolase is secreted and attaches to the cell surface, probably in a complex with plasminogen (Plg) to assist in microbial dissemination in hosts [16,18,19,20]. The α-enolases of other streptococcus were recognized as immunodominant antigens [21,22,23], suggesting that this also could be true for *S. iniae* in mice. In this study we identified and characterized a functional α-enolase amino acid sequence homologue, and confirmed that α-enolase is exposed on the surface of *S. iniae*. We further showed that recombinant α-enolase retained its enzymatic activity, and that surface glycolytic enzyme plays a role in plasminogen-binding, cell adhesion/invasion and protecting mice against *S. iniae* infection.

## 2. Results

### 2.1. Molecular Cloning, Expression and Characterization of S. iniae α-Enolase

**Figure 1:**
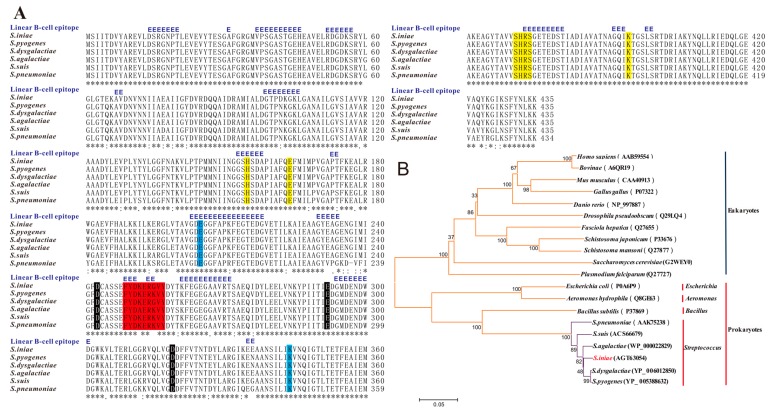
Multiple sequence alignments and phylogenetic analysis of *S. iniae* α-enolase with homologous enolase. (**A**) Alignment of the deduced amino acid sequence of *S. iniae* α-enolase (AGT63054) with those of homologous α-enolase from other *Streptococcus*. These α-enolase sequences were used from *S. dysgalactiae* (YP_006012850; 98%), *S. pyogenes* (YP_005388632; 97%), *S. agalactiae* (WP_000022829; 97%), *S. suis* (ACS66679; 95%), *S. pneumoniae* (AAK75238; 93%), and aligned using the ClustalW2 program. Regions of identity (*), strong similarity (:) and weak similarity (.) were indicated. The putative plasminogen-binding regions were shaded in red. The residues involved in enzyme active sites (205 E, 343 K), metal-binding sites (242 D, 291 E, 318D; magnesium), substrate-binding sites (155 H, 164 E, 291 E, 318 D, 343 K, 370-373 SHRS, 394 K) were shaded in blue, black and yellow, respectively. But there were no any signal peptidase cleavage site or membrane-spanning domains in the amino acid sequence. A residue annotated with a blue “E” was predicted as being part of a linear B-cell epitope (score above threshold 0.7); (**B**) The tree of the α-enolases from the *Streptococcus* of above, some other prokaryotes and eukaryotes was constructed by the neighbour-joining method and plotted with MEGA 4.1. Evolutionary distances were computed using the Poisson correction method. Branch support values (10,000 bootstraps) for nodes were indicated.

Sequence analysis shows that the open reading frame (ORF) of *S. iniae* α-enolase, which is 1308 bp long, encoded a protein of 435 amino acids with a predicted molecular mass 47.24 KDa. A homology search for the protein performed using information obtained from NCBI revealed that *S. iniae* α-enolase shared the highest similarity of amino acid sequence with *S. dysgalactiae* (YP_006012850; 98%), *S. pyogenes* (YP_005388632; 97%), *S. agalactiae* (WP_000022829; 97%), *S. suis* (ACS66679; 95%), and *S. pneumoniae* (AAK75238; 93%) (Figure 1A). Based on the full-length amino acid sequence alignment of the α-enolase protein including the five Streptococcus above, some prokaryotes and eukaryotes, were further determined by phylogenic analysis (neighbour-joining tree) (Figure 1B). The result was in agreement with traditional taxonomy: prokaryotes and eukaryotes were classified in different branches: *Homo sapiens*, *Bovinae*, *Mus musculus*, *Gallus gallus* and *Danio rerio* grouped into a branch and compared with the Streptococcus with remote homology, and all of the Streptococcus sequences grouped into a separate branch. Putative active sites included two enzyme active sites (205 E, 343 K), three metal binding sites (242 D, 291 E, 318 D; magnesium), ten substrate binding sites (155 H, 164 E, 291 E, 318 D, 343 K, 370–373 SHRS, 394 K) and a plasminogen-binding region (248–256 FYDKERKVY) (Figure 1A). Neither signal peptide cleavage sites nor transmembrane helices in the amino acid sequences or proteins were found. Predictive linear B-cell epitope (score above threshold 0.7) were situated in a lot of amino acids residue (Figure 1A).

**Figure 2:**
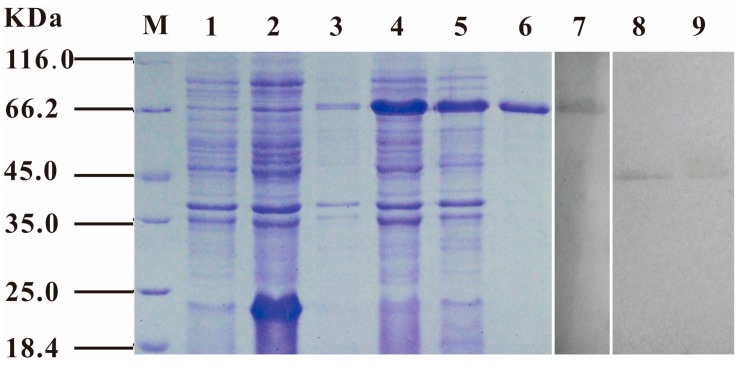
SDS-PAGE and western-blot analysis of *S. iniae* α-enolase. SDS-PAGE was used to analyze the expression of rENO. Recombinant vectors pET32-*eno* and pET32a (+) were transformed into *E. coli* BL21 (DE3) and induced by 0.1 mM IPTG for 4 h at 37 °C. All the samples were analyzed by SDS-PAGE, and the protein was stained with Coomassie Blue R250 in the gel. M, molecular mass marker in KDa; Lane 1–2, the *E. coli* BL21 (DE3) including pET32a (+) was no-induced and induced, respectively; Lane 3, the *E. coli* BL21 (DE3) including pET32-*eno* was no-induced; Lane 4–5, the supernatant and precipitation of the *E. coli* BL21 (DE3) including pET32-*eno* was induced; Lane 6, purified rENO protein. Western-blot was used to analyze the localization of *S. iniae* α-enolase. The rabbit anti-rENO antibodie (1:100) was used to probe the cell wall and cytoplasmic protein fraction of *S. iniae*, and purified rENO was used as control; Lane 7, purified rENO; Lane 8, cell wall protein fraction; Lane 9, cytoplasmic protein fraction.

The *S. iniae* α-enolase ORF was sub-cloned into the pET32a (+) prokaryotic expression vector, and the recombinant protein was overexpressed in *E. coli* BL21(DE3) cells with an N-terminal histidine-tagged fusion protein (~67 KDa). After 3 h of induction in 0.1 mmol/L IPTG, high-level expression of the recombinant protein was observed both in the supernatant and precipitate of the bacteria pyrolysis products (Figure 2, lanes 4–5). After purification by affinity chromatography using His-binding columns under non-denaturing conditions, the rENO proteins were identified by means of SDS-PAGE (Figure 2, lane 6). The purified rENO yield was approximately 1.2 mg/mL from 3.5 L of bacterial culture.

### 2.2. Subcellular Location of S. iniae α-Enolase

To determine the subcellular location of α-enolase in *S. iniae*, purified rabbit anti-rENO serum was analyzed by western-blot to probe the cell wall and cytoplasmic protein fractions of *S. iniae*. Purified rENO was used as a control. Analysis using anti-rENO serum revealed reactivity to a ~47 KDa protein in both the cell walls and cytoplasmic protein fractions (Figure 2, lanes 8–9). The rENO (with an N-terminal Histidine-tagged fusion protein) was revealed at about 67 KDa (Figure 2, lane 7).

### 2.3. Surface Display of S. iniae α-Enolase

**Figure 3:**
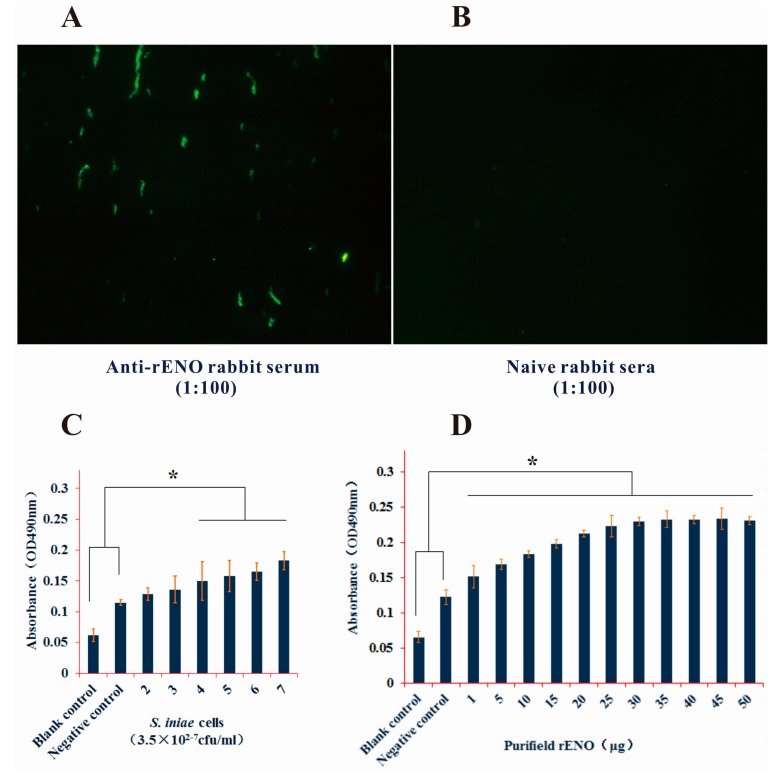
Identification of *S. iniae* α-enolase expression on the cell surface. (**A**,**B**) Indirect immunofluorescence analysis of cell surface α-enolase was carried out with rabbit anti-rENO serum (1:100) or PBS. The fluoresced bacteria cells were showed in chain cocci when bacteria cells were observed by fluorescence microscopy after incubated with FITC conjugated goat anti-rabbit antibody (1:64) and (panel **A**), but the naive rabbit sera control was not observed (panel **B**); (**C**) Rabbit anti-rENO serum was used to detect the surface α-enolase of *S. iniae* by ELISA. The data showed that the absorbance value (OD_450_ nm) increased with the bacteria cells concentration, and the higher concentration bacteria (3.5 × 10^4−6^ cfu/mL) were significant difference compared with negative control (*p* < 0.05); (**D**) Rabbit anti-*S. iniae* whole-cell serum was prepared by our laboratory with formalin inactivated *S. iniae*. The absorbance of rENO incubated with anti-*S. iniae* serum were significant higher than the control incubated with negative serum or PBS (*p* < 0.05). * *p* < 0.05. Error bars indicate SD.

Indirect immunofluorescence analysis using rabbit anti-rENO serum was carried out to assess possible surface localization of α-enolase on *S. iniae*. The results showed that *S. iniae* α-enolase was located on the bacterial cells’ outer surface (Figure 3A,B). Next, we used the rabbit anti-rENO serum to directly detect α-enolase on the cell surface and the rabbit anti-*S. iniae* whole cell serum was used to detect rENO by ELISA. The 96-well plate was coated with different concentrations of *S. iniae* cells. The anti-rENO serum could recognize the α-enolase on the whole cell surface dose dependently (Figure 3C). Significant differences only existed between higher concentrations of bacteria cells and the control (*p* < 0.05). That being said, the data showed that rabbit anti-*S. iniae* whole cells serum included the anti-enolase antibody and had the ability to combine the rENO (Figure 3D). However, our surface staining and ELISA analysis with rabbit anti-rENO serum showed a relatively low signal; this result could be attributed to scarce distribution of α-enolase on the surface as is the case with that of *S. pneumonia*, *S. suis* and *Bacillus anthracis* [18,23,24]. One possible explanation for this could be that other surface structures like proteins might affect the availability of α-enolase epitopes and hinder the reaction with antibody [23]. In addition, environmental conditions were also important factors influencing the transcription and export of α-enolase *in vitro* [25].

### 2.4. α-Enolase Activity and Enzyme Kinetics Analysis

**Figure 4:**
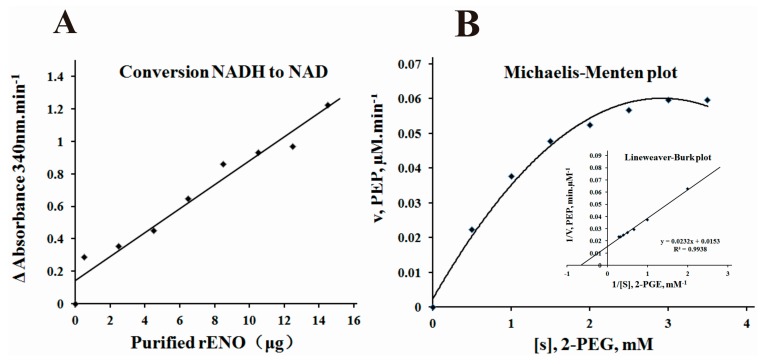
α-enolase activity and enzyme kinetics analysis. (**A**) The coupled-enzyme assay of the rENO was detected by the catalytic rate of conversion of NADH to NAD min^−1^ in the presence of lactate dehydrogenase, ADP, and pyruvate kinase were measured as A_340_ nm·min^−1^; (**B**) Enzyme kinetics of rENO were determined by measuring the rate of conversion of 2-PGE to PEP in the presence of various concentrations of substrate (2-PGE; 0.5–3.5 mM) and 5 μg of the purified rENO and monitored the changes of absorbance at 240 nm. Data was plotted by the method of Michaelis–Menten (inset) and *V*_max_ and *K*_m_ were calculated from double reciprocal plots.

After purification of rENO, it was determined that the recombinant protein possessed the capacity to catalyze the conversion of 2-PGE to PEP. In a coupled-enzyme assay, the rENO protein converted terminal NADH to NAD with dose dependent (Figure 4A). This result indicated the conversion of pyruvate to lactate by lactate dehydrogenase and NADH, confirming the conversion of phosphoglycerate to phosphoenolpyruvate by α-enolase and further conversion to pyruvate with the presence of external pyruvate kinase and ADP. Enzyme reaction kinetics were performed using purified rENO protein with various concentrations of 2-PGE. The Michaelis-Menten plots and double-reciprocal Lineweaver-Burk plots were made to determine *V_max_* and *K_m_* for α-enolase (Figure 4B). This analysis revealed a *K_m_* value of 1.52 mM for 2-PGE and a *V_max_* of 65.36 mM PEP/min for rENO. The *K_m_* observed for PEP, 1.52 mM, falls within the range (1.492 mM for a-enolase from *Streptococcus pyogenes* [26] to 3.3 mM for α-enolase present in *Bacillus anthracis* [18]) reported for enolase from different bacteria.

### 2.5. hPlg Binding Activity of S. iniae α-Enolase

**Figure 5:**
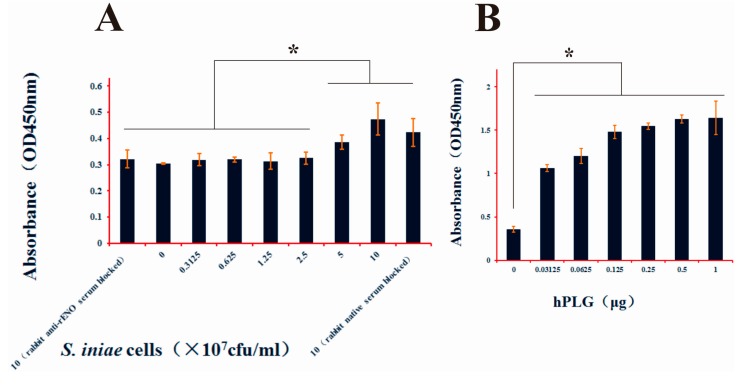
Interaction of human plasminogen with *S. iniae* whole cells and rENO. (**A**) Different concentration of normal *S. iniae* cells (0.3125–10 × 10^7^ cfu/well) and *S. iniae* cells (10 × 10^7^ cfu/well) were blocked by rabbit anti-rENO serum (1:100) or native rabbit serum (1:100) were coated in plates to demonstrate the interaction of human plasminogen (1 μg) with α-enolase of the cells surface. The absorbance values of the high concentration of *S. iniae* cells (5, 10 × 10^7^ cfu/well) and native rabbit serum blocked *S. iniae* cells were significantly higher than other concentration cells and blocked cells (*p* < 0.05); (**B**) The rabbit anti-PLGLA (*N*-term) IgG (1:1000) was used to analyze the direct interaction of the hPLG (0.03125-1 μg) and rENO (1 μg). The binding ability of the rENO to hPLG showed a concentration-dependent increase, and the absorbance values were significant difference compared to the negative control (*p* < 0.05). * *p* < 0.05. NS denotes no statistically significant difference, and error bars indicate SD.

For the ELISA, whole cell bacteria and hPlg were coated on the polystyrene plates. The results demonstrated a concentration dependent increase in binding of *S. iniae* or rENO to hPlg. The statistically difference were significant both in *S. iniae* or rENO compared with the control (*p* < 0.05) (Figure 5A,B). This result confirmed the specificity of the interaction between hPlg and α-enolase on the surface of *S. iniae*.

### 2.6. Adherence and Invasion Assays of S. iniae to BHK-21 Cells

**Figure 6:**
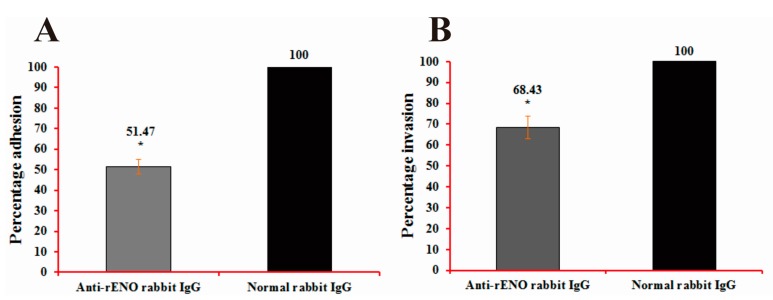
Adhesion (**A**) and invasion (**B**) analyses. Decrease in the adhesion to and invasion of BHK21 cells by *S. iniae* DGX07 through use of rabbit anti-rENO serum. *S. iniae* DGX07 was pre-incubated with the appropriate concentrations of antibodies (1:100) at 37 °C for 60 min prior to infection. The percentage of adhesion to and invasion of cells were 51.47% (*p* < 0.05) and 68.43% (*p* < 0.05) in experimental groups compared to control groups, respectively. Each treatment and control group test was performed in three replicate wells. * *p* < 0.05 in comparison with the level of adhesion/invasion with control antibodies (considered to be 100% adhesion/invasion). Error bars indicate SD.

Adherence is the first step in the pathogenesis of any streptococcal disease, which is carried out by a large number of surface proteins, such as α-enolase. Streptococci can then secrete a great quantity of enzyme and toxin to contribute to their invasion and multiplication by damaging the structure of cells and tissues [8,9]. In order to evaluate the underlying role of α-enolase in *S. iniae* adhesion to and invasion of BHK-21 cells, bacterial α-enolase was blocked with purified rabbit anti-rENO IgG. The results showed that anti-rENO IgG treatment decreased the adhesion of *S. iniae* strain DGX07 to BHK-21 cells to 51.47% (*p* < 0.05) compared with the control (Figure 6A). Invasion of BHK-21 by *S. iniae* was also decreased to 68.43% (*p* < 0.05) compared with the control (Figure 6B). These data show that α-enolase participates in *S. iniae* adhesion to and invasion of BHK-21 cells.

### 2.7. Immunoprotection of rENO as a Subunit Vaccine against S. iniae in Mice

**Figure 7:**
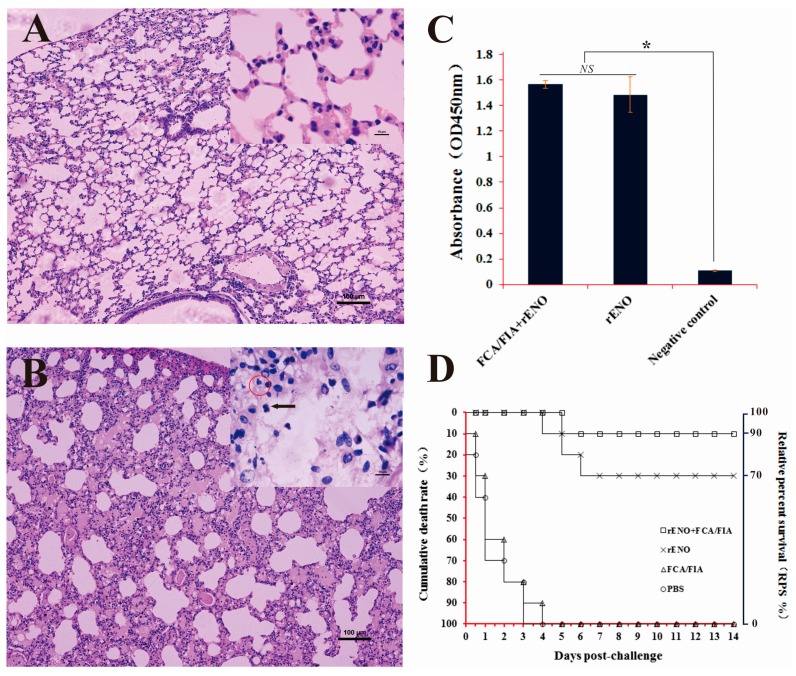
Immunoprotective analysis of rENO against *S. iniae* in mice. (**A**,**B**) Histopathological assessment of lung of immunized mice and control mice. The structure of lungs, were intact and no obvious pathological changes in the immunized group (**A**); However, in the control group, the alveolar walls were thickening and some epithelial cells were shedding; a large number of inflammatory cells infiltrated (as indicated by solid arrow) and the homogeneous red serous exudation led to alveolar lumen narrowing or even disappearance; the bacteria-like structures were observed in macrophages (as indicated by red circle); (**C**) Antibody level analysis of the immune mice. Analysis of total IgG with antiserum 1:100 dilution in response to rENO, the PBS injected mice serum were as the negative control. The antibody levels of immune mice by rENO (with or without adjuvant) were significant higher than the negative control mice (*p* < 0.05). The differences were not significant between rENO immune mice with adjuvant and without adjuvant (*p* > 0.05); (**D**) RPS rates in immunized mice. Mice were immunized two times with rENO-FCA/FIC (□), rENO (×), FCA/FIC (∆) or PBS (O), followed by challenge with *S. iniae* (3.2 × 10^6^ cfu/mice). Each group consisted of 10 mice. All mice were monitored for mortality for 14 days after the challenge, and the cumulative mortality rates (**left** side) corresponding to RPS rates (**right** side) are shown. * *p* < 0.05. NS denotes no statistically significant difference, and error bars indicate SD.

Blood was collected from both immune and control mice two weeks after the last immunization, and antibodies in the serum were assessed by ELISA. The antibody levels in mice immunized by rENO (with or without adjuvant) were significantly higher than the antibody levels in control mice (*p* < 0.05). However, the difference was not significant between rENO immunized mice with adjuvant and without adjuvant (*p* > 0.05) (Figure 7C). It appeared that rENO could induce the mice to produce higher levels of IgG compared to the unimmunized group.

In order to evaluate the efficacy of the rENO protein vaccine against *S. iniae* DGX07 infection, the rENO-immunized mice were challenged intraperitoneally with 3.2 × 10^6^ cfu/mL of *S. iniae*. The tissue lesions in target organs were assessed by histopathology. In the immunized group, the structures of kidney, liver, spleen, brain and lung tissues were intact without obvious pathological changes. In the control group, the alveolar walls were thickened and some of the epithelial cells were shedding. A large number of the inflammatory cells were infiltrated and the homogeneous red serous exudation led to alveolar lumen narrowing or even disappearance; the bacteria-like structures also were observed in macrophages (Figure 7B). Narrowed renal capsules appeared in some of the shed epithelial cells, and inflammatory cell infiltration was observed in glomerulus (data not shown). The livers were severely congested, and there were many inflammatory cells in the blood vessels. The meninges exhibited thickening and hemorrhage (Data not shown).

The control group mice began to die at 12 h after the challenge, whereas the mice in the immunized groups began to die on the fifth or sixth day after the challenge (Figure 7D). The cumulative mortalities of the mice vaccinated with adjuvanted rENO, PBS plus rENO, PBS plus adjuvant and PBS alone were 10%, 30%, 100% and 100%, respectively, which corresponded to a relative survival percentage of 90% or 70% for rENO vaccinated mice with or without adjuvant, respectively (Figure 7D). Altogether, these results confirmed that immunization of mice with recombinant α-enolase effectively protected mice against infection by *S. iniae* DGX07.

## 3. Discussion

*S. iniae* is a major etiological agent of streptococcosis in farmed and wild fish in many regions of the world [27]; it was noted at the 2000 International Conference on Emerging Infectious Diseases as an emerging zoonotic disease transmitted by food animals [28]. In accordance with case reports of humans infected with *S. iniae*, the greatest zoonotic risk appears to be associated with the handling and preparation of infected fish, and the strains isolated from fish and dolphins also showed virulence to humans by phagocytosis assay [3,7]. Our previous studies have confirmed that virulence of *S. iniae* DGX07 (an isolate from farmed channel catfish) in mice was stronger than that in channel catfish [29].

Similar to other virulent and invasive streptococci, such as *S. pyogenes* and *S. pneumoniae*, adhesion and invasion are the key steps in pathogenesis of *S. iniae*. These functions are often performed by a number of surface proteins [30,31], such as α-enolase, a surface exposed moonlighting protein present in a variety of pathogenic microorganisms [17]. We found that the native protein did not only disperse into the cytoplasm and cell walls, but was also secreted to the bacterial cell surface (Figure 2 and Figure 3). The mechanisms of protein exposure to the bacterial cell surface remains unclear, because there are neither signal peptidase cleavage site nor membrane-spanning domains in the deduced amino acid sequence of α-enolase (Figure 1A). So the reassociation of secreted α-enolase to the bacterial cell surface, confirmed by immunoelectron microscopy and binding experiments in *S. pneumonia*, is a potential explanation for surface localization [32].

Previous results suggested that streptococcal surface α-enolase (SEN) has much stronger affinity for direct binding of Plg compared to other described streptococcal surface PLG binding proteins, such as PAM (Plg-binding group A streptococcal M protein) and GAPDH (glyceraldehyde-3-phosphate dehydrogenase) [33,34]. The Plg-enolase interaction is mediated by the C-terminal lysyl residues of *S. pneumonia* α-enolase at position 433 and 434, which have been identified as binding sites for the kringle motifs of Plg [32]. A peptide of nine amino acids (FYDKERKVY) located between amino acids 248–256, was indicated as the minimal second binding epitope mediating binding of Plg to *S. pneumonia* α-enolase [35]. Currently, the two dimensional structure of *S. iniae* α-enolase is not known, however, primary sequence analysis and ClustulW2 alignment has shown that *S. iniae* α-enolase possesses a conserved internal PLG-binding motif (FYDKERGVY) located between amino acids 249 and 257 (Figure 1A). Plg is a single-chain glycoprotein that is inactive until cleaved by Plg activators to form plasmin [36]. The active enzyme consists of five kringle domains, each with three disulfide bonds that contain the lysine binding sites and the catalytic domain. Equipped with proteolytic plasmin activity, pathogenic microorganisms may degrade aggregated fibrin thrombi, thereby promoting the dissemination of pathogens in tissue. The Plg-binding property of many bacteria, notably the Streptococcus species, has been suggested to be a contributing factor in tissue invasion and survival in hosts [18,21,31,37]. Our data suggests that both the surface exposed α-enolase of *S. iniae* and recombinant α-enolase have the ability to bind human Plg (Figure 5). Other studies indicated that bacterial binding of plasminogen can facilitate penetration in different systems. The addition of plasminogen to cultures of *Borrelia burgdorferi* can enhance penetration of endothelial cell monolayers [38]. The interaction between bacterial enolase and plasminogen promotes adherence of *S. pneumoniae* to epithelial and endothelial cells [31]. We found that the rabbit anti-rENO serum treatment decreased the adhesion to and invasion of *S. iniae* DGX07 to the baby hamster kidney-21 (BHK-21) cells compared with the unblocked bacteria cells (Figure 6). These experiments implied that the capture of Plg by surface α-enolase may represent a potent virulence mechanism in *S. iniae* infection hosts.

The surface α-enolase exhibits strong immunogenicity and has been confirmed as an effective vaccine candidate in a variety of pathogenic microorganisms [39,40,41,42]. Recent reports suggested that specific antibodies against α-enolase are likely involved in the protection conferred to *Nile tilapia* by vaccination with the modified *S. iniae* [43] and the recombinant α-enolase also has the ability to protect turbot (*Scophthalmus maximus*) against *S. iniae* infection [44]. In this study, we explored the immunological protection of *S. iniae* α-enolase in mice; immunization with α-enolase significantly increased the specific IgG level of mice compared with the control group. After infected with lethal *S. iniae*, the tissues in the control group displayed severe pathological changes, especially in the lung (Figure 7A), which were not observed in the immunized group with the relative protection ratio above 70% (the relative protection ratio was 90% in the group of rENO plus FCA/FIA). Altogether, these results confirmed that immunization of mice with recombinant α-enolase effectively protects mice against systemic infection by lethal *S. iniae*. Interestingly, rabbit anti-rENO serum can combine with the native α-enolase of both type I and II *S. iniae*, which suggests that α-enolase may have the potential to protect fish against different serotypes *S. iniae* (data not shown).

## 4. Experimental Section

### 4.1. Strains, Plasmids and Media

A highly virulent strain DGX07 of *S. iniae* was isolated from a diseased channel catfish (*Ictalurus punctatus*) [45]. The strain was grown in brain–heart infusion (BHI) broth (Oxoid, Basingstoke, UK) in an airtight conical flask without agitation at 37 °C [46]. *Escherichia coli* strains DH5α and BL21 (DE3) (Invitrogen, Carlsbad, CA, USA) were used for cloning and expression experiments, respectively. *E. coli* strains were grown in Luria-Bertani (LB) broth or on agar plates at 37 °C. Ampicillin (100 μg/mL; Sangon Biotech, Shanghai, China) was used in growth media when required. The vectors pMD19-T (Takara, Dalian, China) and pET-32a (+) (Invitrogen, Carlsbad, CA, USA) were used for polymerase chain reaction (PCR) cloning and protein expression *in vitro*, respectively.

### 4.2. Animals

This study was reviewed and approved by the Animal Ethics Committee of Sichuan Agricultural University (Ya’an, China; Approval No. 2011–028). New Zealand white rabbits and six week-old male specific-pathogen-free (SPF) BALB/c mice were purchased from the Laboratory Animal Center of Sichuan University. All animals were housed under a barrier environment in sterile cages in the laboratory animal house (ambient temperature of 21–25 °C, humidity of 40%–60%, and a 12-h light/dark cycle) and fed pelleted food and sterilized water *ad libitum*. Animals were acclimated to these conditions for one week prior to the experiment. All surgical procedures were performed under isoflurane anesthesia and all mice were sacrificed by cervical dislocation. All efforts were made to minimize suffering.

### 4.3. Amplification and Bioinformatics Analyses

Total DNA for *S. iniae* DGX07 was used as the template for PCR amplification with a sense primer (5′-ATGTCAATTATTACTGATG-3′) and an antisense primer (5′-TTATTTTTTTAGGTTGTAG-3′) designed to target the *S. iniae* 9117 genome sequence (GenBank accession: AMOO01000001). The PCR amplified product was gel-purified, cloned into the pMD19-T vector and sequenced. The Lasergene software package for Windows (DNASTAR, Madison, WI, USA) was used to analyze the open reading frame (ORF) of the nucleotide sequence and deduce the amino acid sequence. The sequence was assigned GenBank accession number AGT63054 from the National Center for Biotechnology Information (NCBI). Similarity comparisons with previously reported sequences in GenBank were performed using DNAMAN version 3.0 (Lynnon Biosoft, Vaudreuil, QC, Canada) and on-line Blast tools at the NCBI website (http://blast.ncbi.nlm.nih.gov/Blast.cgi). Based on their similarities, multiple sequence alignment by ClustalW2 (http://www.ebi.ac.uk/Tools/clustalw2/index.html), and phylogenetic analysis was built using the neighbour-joining (NJ) method with 10,000 bootstrapping replications of the Mega 4.1 program. The conserved active sites of *S. pneumonia* Q97QS2 (ENO_STRPN) (UniProtKB/Swiss-Prot) α-enolase protein were used as a reference to *S. iniae* α-enolase. The signal peptide cleavage sites and transmembrane helices were predicted by SignalP 4.1 Server (http://www.cbs.dtu.dk/services/SignalP/) and TMHMM Server v. 2.0 (http://www.cbs.dtu.dk/services/TMHMM/), respectively. In addition, in order to further investigate the possible mechanism underlying the strong immunogenicity of *S. iniae* α-enolase, we conducted B cell epitope prediction using BepiPred, version 1.0 (http://www.cbs.dtu.dk/services/BepiPred).

### 4.4. Cloning, Expression, and Purification of S. iniae α-Enolase

The coding sequence of α-enolase was amplified by PCR using a sense primer (5′-CGCGGATCCATGTCAATTATTACTGATG-3′) and an antisense primer (5′-CGGAAGCTTCTATTTTTTTAGGTTGTAG-3′) with *BamH*I and *Hind*III restriction enzyme sites (underlined), respectively. After pMD19-T cloning and direct sequencing, *eno* was inserted into pET32a (+) via digested *BamH*I and *Hind*III, generating pET32-*eno*. The pET32-*eno* was transformed into *E. coli* BL21 (DE3) and was induced by isopropyl-β-d-thiogalactopyranoside (IPTG) to express protein at a final concentration of 0.1 mM for 4 h at 37 °C. Bacterial cells were collected from the solution by centrifugation and resuspended in Ni-Native-0 buffer (50 mM NaH_2_PO_4_, 300 mM NaCl, pH 8.0). The resuspended bacterial cells were broken up by sonication and the supernatant that may contain soluble protein was collected by centrifugation at 12,000 rmp at 4 °C for 20 min, then the supernatant with Ni-Native-0 buffer was added into the equilibrated Ni-NTA-Sefinose column (Sangon Biotech, Shanghai, China). Then, the resin-absorbed histidine-tagged fusion protein was washed using Ni-Native-250 buffer (50 mM NaH_2_PO_4_, 300 mM NaCl, 250 mM imidazole, pH 8.0). The purified product was analyzed by means of 12.5% sodium dodecyl sulphate polyacrylamide gel electrophoresis (SDS-PAGE). Final concentrations of purified proteins were measured with the micro-BCA protein assay reagent (Sangon Biotech, Shanghai, China), and any potential endotoxins were removed by the EndotoxinOUT™ Resin (Sangon Biotech, Shanghai, China).

### 4.5. Preparation of Anti-rENO Serum

To obtain rabbit polyclonal sera against rENO, two male New Zealand white rabbits were immunized with a subcutaneous injection of 1 mg of rENO purified as described above and mixed with Freund complete adjuvant (1:1) (Sigma, St. Louis, MO, USA). After 2 weeks each rabbit received a booster injection with the same antigen concentration emulsified with Freund incomplete adjuvant (1:1) (Sigma, St. Louis, MO, USA). A second booster injection was administered three days after the first. Serum samples were collected three days after the second booster injection. All surgical procedures were performed under isoflurane anesthesia.

### 4.6. Isolation of Cellular Protein Fractions

In order to confirm the location of *S. iniae* α-enolase, cytosolic and cell wall fractions of *S. iniae* DGX07 were isolated as described by Jones and Holt, with some modifications [37]. Briefly, the *S. iniae* DGX07 cells were harvested by centrifugation (12,000× *g*) from 50 mL of cultures grown 48h without agitation at 37 °C in BHI broth. The cells were washed twice with 20 mM phosphate buffered saline (PBS, pH 7.0), and resuspended in the same PBS. The cell suspension was ruptured with a Mini-Beadbeater using 0.1 mm silica beads for 3 min. Then the supernatant (total cell extract) was removed from the mixture. Cytoplasmic protein fractions were isolated by centrifugation (48,000× *g* for 45 min, 4 °C) from the final supernatant and the pellet comprised of cell wall protein fractions was resuspended in PBS. The cellular protein fraction solution was stored in small aliquots at −80 °C.

### 4.7. SDS-PAGE and Western-Blot Analysis

SDS-PAGE and western-blot analyses were performed as described by Xie *et al.* [47]. Protein samples were separated in 12.5% SDS-PAGE and electrophoretically transferred onto PVDF membranes. The membranes were incubated for 2 h in Tris-Buffered Saline with Tween-20 (TBST) containing 3% Bovine Serum Albumin (BSA, Sangon Biotech, Shanghai, China). To separate the α-enolase from the cytoplasmic and cell wall protein fractions, anti-rENO rabbit serum and naive rabbit serum (negative control) were used. After three washes with TBST, the membranes were further incubated for 2 h with 1:5000 diluted HRP goat anti-Rabbit IgG Antibody (ABGENT, San Diego, CA, USA). Protein signals were visualized with the DAB Substrate Kit (20×) (ABGENT, San Diego, CA, USA).

### 4.8. Indirect Immunofluorescence Assay

The α-enolase on the bacterial cell surface was detected by indirect immunofluorescence assay as described elsewhere [18]. Briefly, the *S. iniae* was grown to an OD_600_ of 0.6 in BHI broth. One mL of the cell suspension was washed three times with PBS and fixed in solution (acetic acid and methanol in 1:3 ratio). Samples were stored at 4 °C for 1 h and dropped on pre-cleaned, chilled glass slides, which were then air-dried and washed three times with PBS. Cells coated on the glass slides were incubated for 2 h with a 1:100 dilution of anti-rENO rabbit serum or naive rabbit serum (negative control) at 37 °C, followed by three washes with PBS. Samples incubated for 1 h at 37 °C after addition of fluorescein isothiocyanate (FITC) conjugated goat anti rabbit IgG (1:64; ABGENT, USA) to each sample. After incubation, samples were mounted in a solution containing glycerol and PBS in a 1:1 ratio. The slides were examined using a fluorescence microscope (Eclipse 80i, Nikon, Tokyo, Japan).

### 4.9. ELISA Analysis

ELISA was performed to test for the presence of α-enolase on the surface of *S. iniae*. In reference to the method described above, 100 μL of differing concentrations of *S. iniae* cells (3.5 × 10^2−7^ cfu/mL) or purified rENO (1–50 μg) were coated in 96-well plates. Control wells were coated with the highest concentration of cells or protein. The plates were incubated for 16 h at 4 °C and 1:100 diluted rabbit anti-rENO serum or rabbit anti-formalin inactivated whole cell serum of *S. iniae* (prepared by Defang Chen of our laboratory) was used to detect α-enolase. After adding 1:2000 dilution of HRP Goat anti-Rabbit IgG (ABGENT, USA), TMB (Tiangen, Beijing, China) was used to stain the samples, and absorbance was measured at 450 nm in a microplate ELISA reader (Bio-Rad, Hercules, CA, USA).

### 4.10. Determination of α-Enolase Activity

**Figure 8:**
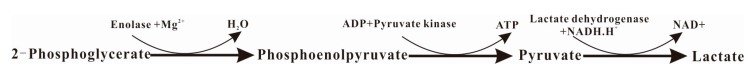
The reactions of transformation of NADH.H^+^ to NAD^+^ associate with a-enolase.

The a-enolase activity was measured as described by Pancholi and Fischetti for both the coupled and the direct assays [48]:

(i) Coupling analysis. The a-enolase activity was determined by measuring the transformation of NADH.H^+^ to NAD^+^ (Figure 8).

The enzymatic reactions were performed at room temperature in 100 mM HEPES buffer (pH 7.0, containing 3.3 mM MgSO_4_, 0.2 mM NADH, 1.0 mM 2-PGE, 1.2 mM ADP, 10.3 IU of lactate dehydrogenase, and 2.7 IU of pyruvate kinase in a final reaction volume of 1.0 mL; Sigma, USA), and the total volume was 1 mL in a 1 cm quartz cuvette. The decrease in absorbance at 340 nm was recorded as the change per minute by continuous spectrophotometer CE-1021 (Cecil, Cambridge, UK).

(ii) Enzyme kinetics analysis. To study α-enolase enzyme kinetics, different amounts of 2-PGE (0.5–3.5 mM) were pre-incubated at room temperature for 3 min using 5 μg of purified α-enolase protein in HEPES buffer (100 mM HEPES, 10 mM MgCl_2_, and 7.7 mM KCl; pH, 7.0). The release of PEP was measured at 240 nm on a continuous spectrophotometer CE-1021 (Cecil, Cambridge, UK). Michaelis-Menten kinetics of rENO showed that rENO was able to convert 2-PGE to PEP for all substrate concentration levels. *V_max_* and *K_m_* for rENO were determined from double-reciprocal Lineweaver-Burk plots.

### 4.11. Plasminogen Interaction of α-Enolase

*S. iniae* cells prepared from overnight cultures (10, 5, 2.5, 1.25, 0.625, 0.3125 × 10^7^ cfu/well) or *S. iniae* cells (10 × 10^7^ cfu/well) that were blocked by rabbit serum (1:100, anti-rENO rabbit serum or native rabbit serum) or human plasminogen (1, 0.5, 0.25, 0.125, 0.0625, 0.03125 μg, hPlg; Sigma, USA), in 50 mM carbonate coating buffer at pH 9.6, were used to coat 96-well plates. After incubating the cells for 16 h at 4 °C in triplicates, the wells were washed with phosphate buffer saline (PBS) containing 0.05% Tween 20 (PBST) and blocked with 100 μL of 3% BSA-PBST for 1 h at 37 °C. One μg of hPlg was added in the wells containing immobilized bacteria to assess the interaction of *S. iniae* cells with hPlg. One μg of rENO was added to the wells containing hPlg to assess the direct interaction between hPlg and rENO. After incubation for 1 h at 37 °C the wells were washed thrice, and 100 μL rabbit anti-PLGLA (*N*-term) IgG (1:1000; ABGENT, San Diego, CA, USA) or mouse anti-His Tag IgG (1:1000; ABGENT, San Diego, CA, USA) was added, and incubated for 1 h at 37 °C, respectively. Subsequent incubation was followed with 1:2000 dilution of HRP goat anti-rabbit IgG and HRP goat anti-mouse IgG for 1 h at 37 °C, respectively. The color was developed by adding 100 μL TMB (Tiangen, Beijing, China) and absorbance was measured at 450 nm in a microplate ELISA reader (Bio-Rad, Hercules, CA, USA).

### 4.12. Adherence and Invasion Assays

Baby hamster kidney-21 (BHK-21) cells were grown to confluence in 24-well tissue culture plates (1–5 × 10^5^ cells/well) and washed with DMEM high glucose medium without fetal calf serum and antibiotics. *S. iniae* from a mid-log-phase culture (~1 × 10^7^ cfu/mL) were incubated with equal concentrations of either native rabbit IgG or purified rabbit anti-rENO IgG for 1 h at 37 °C in fresh DMEM medium without antibiotics. Confluent cell monolayers were inoculated with 1 mL aliquots of either bacterial suspension. Following centrifugation at 350× *g* for 5 min, the plate was incubated for 30 min at 37 °C with 5% CO_2_. The cells were washed five times with DMEM, then three times with PBS, and lysed by adding 100 μL of 0.01% Triton X-100 (Sigma, St. Louis, MO, USA). The adherent bacteria were quantified by plating serial dilutions of lysed cell suspension on BHI. Invasion assays were carried out in a similar manner except that the bacteria were incubated with the cells for 1 h. After washing the cells three times, the samples were incubated in fresh DMEM with a 100 U/mL penicillin streptomycin combination (Sangon Biotech, Shanghai, China) for 2 h to kill extracellular bacteria. Cells were washed three times and lysed by Triton X-100 prior to enumeration of CFU. Each treatment and control group were performed in three replicate wells.

### 4.13. Immune Protection Assay

For immunization, rENO proteins were diluted in PBS (0.01 M, pH 7.4) at a concentration of 500 μg/mL and mixed with an equal volume of Freund’s complete adjuvant (FCA) or Freund’s incomplete adjuvant (FIA) (Sigma, St. Louis, MO, USA) as described previously [39]. Control FCA or FIA mixed with PBS and control PBS were also used for challenge studies. Eighty mice were randomly assigned to four groups and injected subcutaneously with the rENO mixed with FCA, rENO mixed with PBS, FCA mixed with PBS or PBS alone (200 μL per animal). The booster injections were prepared in the same method and dosage at 14-day intervals after the first injection, but the FCA was replaced by FIA. Five mice from each group were sacrificed two weeks after the final booster injection, and the serum was removed aseptically for rENO-specific serum IgG antibody measurements. The remaining 15 animals in each group were injected with 3.2 × 10^6^ cfu/mouse *S. iniae* (tenfold LD_50_) via the intra-abdominal cavity, as previously described by Chen [29]. Five mice were sacrificed on day 7 after infection as for histopathological assessment, and the dying mice infected by *S. iniae* (confirmed by bacteria isolation and identification from kidney and lung) were also collected for histopathological observation. The brains, kidneys, livers, spleens and lungs were removed, suspended in 10% (*v*/*v*) formalin for 24 h, dehydrated, and embedded in paraffin. Section 3 and Section 4 μm-thick were prepared, stained with Hematoxylin and Eosin [39], and observed under a light microscope (Nikon, Tokyo, Japan). For the last 10 remaining mice in each group, mortality was monitored over a period of 14 days after the challenge, and dying mice were randomly selected for examination of bacterial recovery from the liver, kidney and spleen as described by Zhang *et al.* [49]. Relative percent of survival (RPS) was calculated as follows: RPS = (1 − (% mortality in immunized mice/% mortality in control mice)) × 100 [50].

### 4.14. Statistical Analysis

The data were expressed as the mean ± standard deviation (SD). Comparisons between experimental groups were performed by one-way ANOVA. LSD and Duncan’s test using SPSS17.0 Data Editor (SPSS Inc., Chicago, IL, USA). *p* values < 0.05 were considered to be significant.

## 5. Conclusions

The data presented here illustrated that the native α-enolase protein did not only distribute in the cytoplasm, but also can be secreted to the *S. iniae* cell surface. The surface α-enolase participates in *S. iniae* adhesion to and invasion of BHK-21 cells and has affinity for binding of hPlg, and it exhibits potential as an effective subunit vaccine against *S. iniae* infections in mice. However, how the pathogen utilizes the Plg-enolase interaction to promote *S. iniae*’s adhesion to host cells and invasion of tissue is not clear, and whether the α-enolase produces cross protection effects to different serotypes of *S. iniae* in fish or mammals, are both questions that need to be investigated in further studies.
